# Supplementary figures and images for: The G protein alpha chaperone and guanine-nucleotide exchange factor RIC-8 regulates cilia morphogenesis in Caenorhabditis elegans sensory neurons
Source: PLoS Genet. 2023 Nov 1;19(11):e1011015. doi: 10.1371/journal.pgen.1011015 (PMC10642896; doi:10.1371/journal.pgen.1011015)

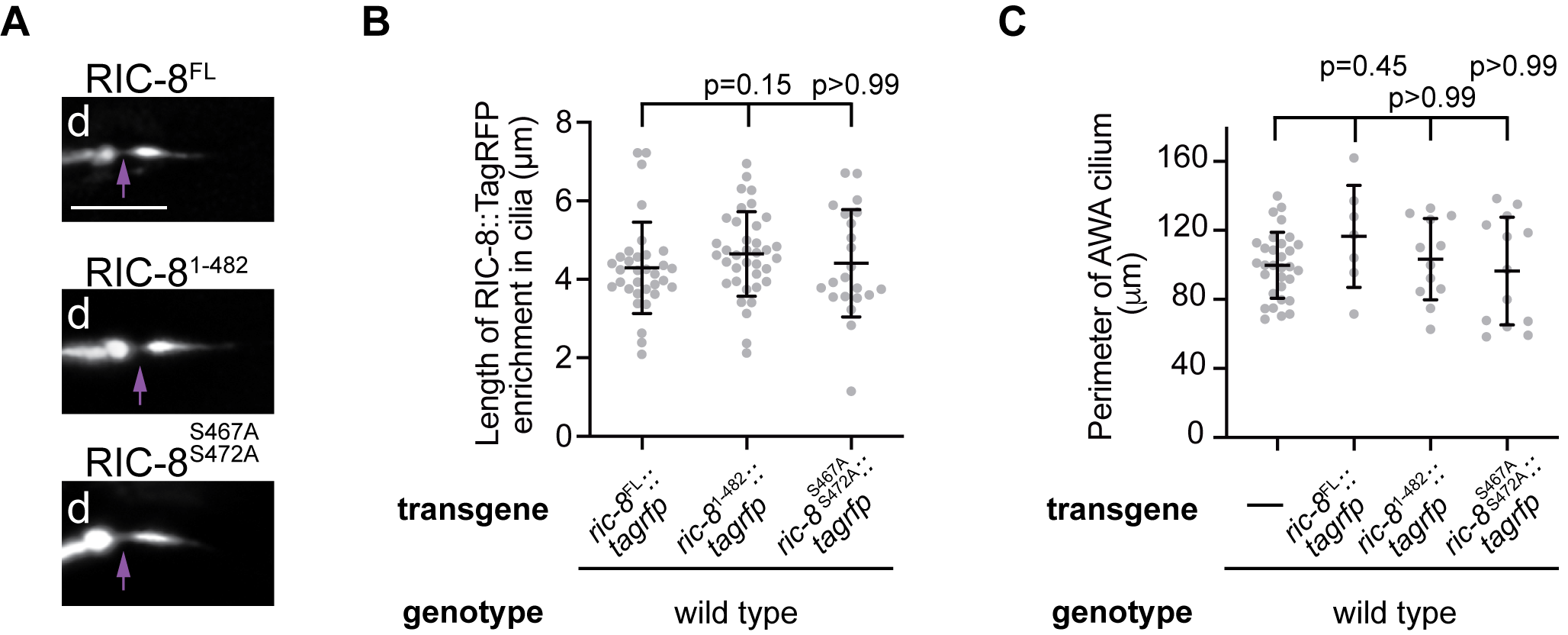

Supplement: S1 Fig — (A–B) Representative images (A) and quantification (B) of localization patterns for the indicated RIC-8::TagRFP constructs in cilia of phasmid neurons. Anterior is at left; d–dendrite; purple arrowheads mark cilia base; scale bar: 5 μm. (C) Quantification of AWA cilium perimeter in adult animals of the indicated genotypes. Data for wild type are repeated from Fig 3C. In all scatter plots, means ± SD are indicated by horizontal and vertical black bars, respectively. The p-values were calculated using Kruskal-Wallis with Dunn’s multiple comparisons test. (TIF) [file pgen.1011015.s001.tif]

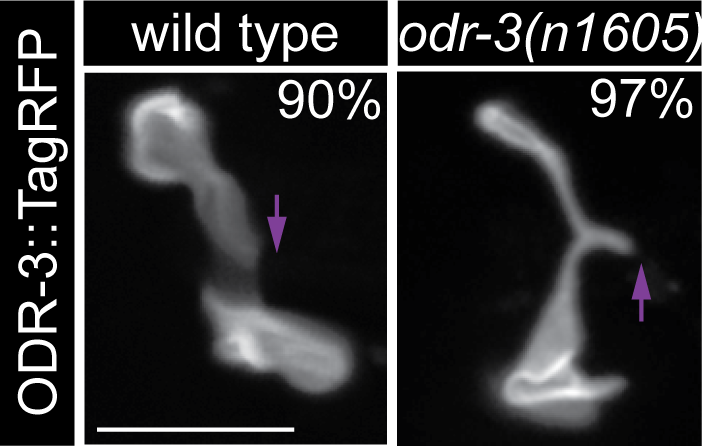

Supplement: S2 Fig — Representative images of ODR-3∷TagRFP localization in AWC cilia of wild-type and odr-3(n1605) mutant adults. Numbers in top right corners indicate percentage of animals exhibiting the depicted phenotype (n>30/genotype). Anterior is at left; purple arrowheads mark cilia base; scale bar: 5 μm. (TIF) [file pgen.1011015.s002.tif]

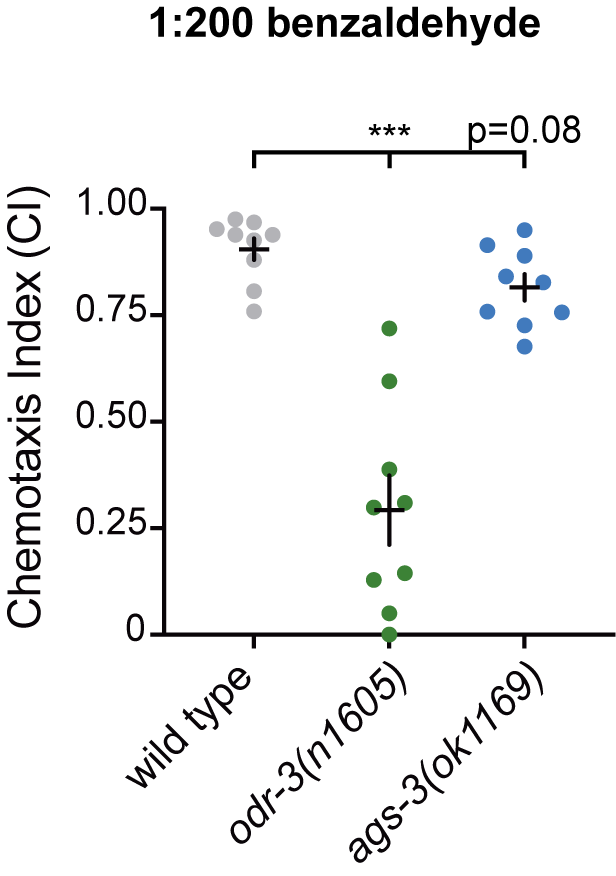

Supplement: S3 Fig — Chemotaxis responses of wild-type, odr-3(n1605), and ags-3(ok1169) adult hermaphrodites to benzaldehyde diluted in ethanol (1:200). Dots–CI from single assays of approximately 200 animals each. Means ± SEM are indicated by horizontal and vertical black bars, respectively. *** indicates different from wild type at p < 0.001 (Brown-Forsythe and Welch ANOVA with Dunnett’s T3 multiple comparisons test). (TIF) [file pgen.1011015.s003.tif]
