# Supplementary material for: The G protein alpha chaperone and guanine-nucleotide exchange factor RIC-8 regulates cilia morphogenesis in Caenorhabditis elegans sensory neurons
Source: PLoS Genet. 2023 Nov 1;19(11):e1011015. doi: 10.1371/journal.pgen.1011015 (PMC10642896; doi:10.1371/journal.pgen.1011015)
Supplement: S1 Table — (DOCX) [file pgen.1011015.s004.docx]

**S1 Table:** List of *C. elegans* strains used in this work

| **Strain** | **Genotype** | **Source** |
| --- | --- | --- |
| PY1089 | *kyIs104[str-1*p*::gfp]* X | (1) |
| NWM017 | *ric-8(md303)* IV; *kyIs104[str-1*p*::gfp]* X | This work |
| NWM272 | *ric-8(md1909)* IV; *kyIs104[str-1*p*::gfp]* X | This work |
| NWM089 | *ric-8(ok98)/nT1[qIs51]* IV; *kyIs104[str-1*p*::gfp]* X | This work |
| PSAB1120 | *oyIs88[gpa-4Δ6*p*∷myr-gfp]* | (2) |
| PY3453 | *oyIs50[ceh-36*p*::gfp]* IV | (3) |
| NWM058 | *ric-8(ok98)/nT1[qIs51]* IV; *oyIs88[gpa-4Δ6*p*∷myr-gfp]* | This work |
| NWM059 | *ric-8(ok98)/nT1[qIs51]* IV; *nchEx015[odr-1*p*::rfp]* | This work |
| NWM237 | *ric-8(md1909)* IV; *oyIs50[ceh-36*p*::gfp]* IV | This work |
| NWM386 | *ric-8(md1909)* IV; *oyIs50[ceh-36*p*::gfp]* IV; *nchEx001[ceh-36Δ*p*∷ric-8∷tagrfp, unc-122Δ*p*∷dsRed]* | This work |
| NWM169 | *ric-8(md303)* IV; *nuIs11[osm-10*p*∷gfp + lin-15(+)]* | This work |
| HA3 | *nuIs11[osm-10*p*::gfp + lin-15(+)]* | CGC |
| NWM108 | *nchEx002[bbs-8*p*::myrgfp, bbs-8*p*::ric-8::tagrfp, unc-122Δ*p*::gfp]* | This work |
| NWM393 | *nchEx003[bbs-8*p*::ric-8::tagrfp, bbs-8*p*::nphp-2s::gfp, unc-122Δ*p*::dsRed]* | This work |
| NWM344 | *oyIs50[ceh-36*p*::gfp]* IV; *nchEx001[ceh-36Δ*p*∷ric-8∷tagrfp, unc-122Δ*p*∷dsRed]* | This work |
| NWM031 | *nchEx004[bbs-8*p*∷ric-8∷tagrfp, unc-122Δ*p*∷gfp]*; *nchEx013[nphp-4*p*∷nphp-4∷gfp, unc-122Δ*p*∷dsRed]* | This work |
| NWM036 | *ric-8(md303)* IV; *oyIs88[gpa-4Δ6*p*∷myr-gfp]* | This work |
| NWM255 | *ric-8(md1909) IV; oyIs88[gpa-4Δ6*p*∷myr-gfp]* | This work |
| NWM133 | *ric-8(md303)* IV; *oyIs88[gpa-4Δ6*p*∷myr-gfp]*; *nchEx004[bbs-8*p*∷ric-8∷tagrfp, unc-122Δ*p*∷gfp]* | This work |
| NWM187 | *ric-8(md303)* IV; *oyIs88[gpa-4Δ6*p*∷myr-gfp]*; *nchEx005[bbs-8*p*∷ric-8^1-483^∷tagrfp, unc-122Δ*p*∷gfp]* | This work |
| NWM260/  NWM261 | *ric-8(md1909)* IV; *oyIs88[gpa-4Δ6*p*∷myr-gfp]*; *nchEx006[bbs-8*p*∷ric-8^1-522^∷tagrfp, unc-122Δ*p*∷gfp]* | This work |
| NWM236 | *ric-8(md303)* IV; *oyIs88[gpa-4Δ6*p*∷myr-gfp]*; *nchEx007[bbs-8*p*∷ric-8^S467A,S472A^∷tagrfp, unc-122Δ*p*∷gfp]* | This work |
| NWM525 | *oyIs88[gpa-4Δ6*p*∷myr-gfp]*; *nchEx004[bbs-8*p*∷ric-8∷tagrfp, unc-122Δ*p*∷gfp]* | This work |
| NWM219/  NWM220 | *oyIs88[gpa-4Δ6*p*∷myr-gfp]*; *nchEx005[bbs-8*p*∷ric-8^1-483^∷tagrfp, unc-122Δ*p*∷gfp]* | This work |
| NWM226 | *oyIs88[gpa-4Δ6*p*∷myr-gfp]*; *nchEx007[bbs-8*p*∷ric-8^S467A,S472A^∷tagrfp, unc-122Δ*p*∷gfp]* | This work |
| NWM185 | *oyIs50[ceh-36*p*∷gfp]* IV; *odr-3(n1605)* V | This work |
| NWM286 | *oyIs50[ceh-36*p*∷gfp]* IV; *ric-8(md1909)* IV; *odr-3(n1605)* V | This work |
| NWM313/  NWM390 | *oyIs50[ceh-36*p*∷gfp]* IV; *nchEx008[ceh-36Δ*p*∷odr-3∷tagrfp, unc-122Δ*p*∷gfp]* | This work |
| NWM262/  NWM243 | *oyIs50[ceh-36*p*∷gfp]* IV; *odr-3(n1605)* V; *nchEx008[ceh-36Δ*p*∷odr-3∷tagrfp, unc-122Δ*p*∷gfp]* | This work |
| NWM292 | *nchEx009[hsp-16.2*p*∷ric-8∷vc155, ceh-36Δ*p*∷odr-3∷vn173, unc-122Δ*p*∷dsRed]* | This work |
| NWM273/  NWM274 | *nchEx010[hsp-16.2*p*∷vc155, ceh-36Δ*p*∷vn173, unc-122Δ*p*∷dsRed]* | This work |
| NWM412 | *nchEx011[hsp-16.2*p*∷ric-8^1-483^∷vc155, ceh-36Δ*p*∷odr-3∷vn173, unc-122Δ*p*∷dsRed]* | This work |
| NWM413 | *oyIs50[ceh-36*p*∷gfp]* IV; *ric-8(md1909)* IV; *nchEx008[ceh-36Δ*p*∷odr-3∷tagrfp, unc-122Δ*p*∷gfp]* | This work |
| NWM397 | *nchEx012[ceh-36Δ*p*∷ric-8∷gfp, unc-122Δ*p*∷dsRed]* | This work |
| NWM430 | *odr-3(n1605)* V; *nchEx012[ceh-36Δ*p*∷ric-8∷gfp, unc-122Δ*p*∷dsRed]* | This work |
| NWM302 | *oyIs50[ceh-36*p*∷gfp]* IV; *ags-3(ok1169)* X | This work |
| NWM396 | *oyIs50[ceh-36*p*∷gfp]* IV; *ric-8(md1909)* IV; *ags-3(ok1169)* X | This work |
| NWM482/  NWM483 | *oyIs50[ceh-36*p*∷gfp]* IV; *ags-3(ok1169)* X; *nchEx008[ceh-36Δ*p*∷odr-3∷tagrfp, unc-122Δ*p*∷dsRed]* | This work |
| NWM454/  NWM522 | *ric-8(md1909)* IV; *oyIs50[ceh-36*p*::gfp]* IV; *ags-3(ok1169)* X; *nchEx008[ceh-36Δ*p*∷odr-3∷tagrfp, unc-122Δ*p*∷dsRed]* | This work |
| N2 | *C. elegans* wild isolate | CGC |
| CX3222 | *odr-3(n1605)* V | CGC |
| RB1145 | *ags-3(ok1169)* X | CGC |
| NWM431 | *oyIs50[ceh-36*p*::gfp]* IV; *nchEx014[ceh-36Δ*p*∷grk-2^CT^, unc-122Δ*p*∷dsRed]* | This work |
| NWM455 | *ric-8(md1909)* IV; *oyIs50[ceh-36*p*::gfp]* IV; *nchEx014[ceh-36Δ*p*∷grk-2^CT^, unc-122Δ*p*∷dsRed]* | This work |
| NWM352 | *oyIs50[ceh-36*p*∷gfp] IV; nchEx016[ceh-36Δ*p*∷odr-3^Q206L^, unc-122Δ*p*∷dsRed]* | This work |

1. Troemel ER, Kimmel BE, Bargmann CI. Reprogramming Chemotaxis Responses: Sensory Neurons Define Olfactory Preferences in C. elegans. Cell. 1997;91(2):161-9.

2. Maurya AK, Rogers T, Sengupta P. A CCRK and a MAK Kinase Modulate Cilia Branching and Length via Regulation of Axonemal Microtubule Dynamics in Caenorhabditis elegans. Current Biology. 2019;29(8):1286-300.e4.

3. Kim K, Kim R, Sengupta P. The HMX/NKX homeodomain protein MLS-2 specifies the identity of the AWC sensory neuron type via regulation of the ceh-36 Otx gene in C. elegans. Development. 2010;137(6):963-74.

**References**
