# Supplementary material for: The G protein alpha chaperone and guanine-nucleotide exchange factor RIC-8 regulates cilia morphogenesis in Caenorhabditis elegans sensory neurons
Source: PLoS Genet. 2023 Nov 1;19(11):e1011015. doi: 10.1371/journal.pgen.1011015 (PMC10642896; doi:10.1371/journal.pgen.1011015)
Supplement: S2 Table — (DOCX) [file pgen.1011015.s005.docx]

| **Plasmid** | **Description** | **Source** |
| --- | --- | --- |
| NWM017 | *ceh-36Δ*p*∷ric-8∷tagrfp* | This work |
| Co-injection marker | *unc-122Δ*p*∷dsRed* | (1) |
| NWM016 | *bbs-8*p*::myr-gfp* | This work |
| NWM005 | *bbs-8*p*::ric-8::tagrfp* | This work |
| Co-injection marker | *unc-122Δ*p*::gfp* | (1) |
| NWM047 | *bbs-8*p*::nphp-2s::gfp* | This work |
| NWM010 | *bbs-8*p*∷ric-8^1-483^∷tagrfp* | This work |
| NWM029 | *bbs-8*p*∷ric-8^1-522^∷tagrfp* | This work |
| NWM007 | *bbs-8*p*∷ric-8^S467A,S472A^∷tagrfp* | This work |
| NWM032 | *ceh-36Δ*p*∷odr-3∷tagrfp* | This work |
| NWM030 | *hsp-16.2*p*∷ric-8∷vc155* | This work |
| NWM034 | *ceh-36Δ*p*∷odr-3∷vn173* | This work |
| NWM031 | *hsp-16.2*p*∷vc155* | This work |
| NWM033 | *ceh-36Δ*p*∷vn173* | This work |
| NWM046 | *hsp-16.2*p*∷ric-8^1-483^∷vc155* | This work |
| NWM043 | *ceh-36Δ*p*∷ric-8∷gfp* | This work |
| NWM048 | *ceh-36Δ*p*∷grk-2^CT^* | This work |
| NWM040 | *ceh-36Δ*p*∷odr-3^Q206L^∷tagrfp* | This work |

**S2 Table:** List of plasmids used in this work

1. Miyabayashi T, Palfreyman MT, Sluder AE, Slack F, Sengupta P. Expression and function of members of a divergent nuclear receptor family in Caenorhabditis elegans. Dev Biol. 1999;215(2):314-31.

**REFERENCES**
