# Supplementary material for: The G protein alpha chaperone and guanine-nucleotide exchange factor RIC-8 regulates cilia morphogenesis in Caenorhabditis elegans sensory neurons
Source: PLoS Genet. 2023 Nov 1;19(11):e1011015. doi: 10.1371/journal.pgen.1011015 (PMC10642896; doi:10.1371/journal.pgen.1011015)
Supplement: Attachment — Submitted filename: Response to Reviewers.pdf [file pgen.1011015.s007.pdf]

Responses to Reviewers
We would like to thank the reviewers for their thoughtful and insightful comments that helped us improve the quality and clarity of the manuscript. Our responses to all reviewers' questions are summarized below.

Please note: the line number references are for the revised manuscript version that does not include tracked changes.

Reviewer #1:

1. *RIC-8 localization: Authors convincingly show that RIC-8 co-localizes with Inversin/NPHP-2S to the proximal region of rod cilia but not wing cilia. Do wing cilia possess an inversin compartment? What do authors think RIC-8 is doing in the inversin compartment of rod cilia? This would be an appropriate discussion point.*

The organization or composition of the inversin compartment has not been directly examined in the individual wing neurons. However, it has been previously reported that certain signaling molecules that are present in the inversin compartment of the channel cilia (e.g. ARL-13, TAX-4, CNG-3) are distributed throughout the wing cilia (Cevik et al., 2010 PMID: 20231383; Wojtiniak et al., 2013 PMID: 23886944). RIC-8 exhibits a similar localization pattern suggesting that it may play a role in regulating signaling pathways that are restricted to the inversin compartment of rod cilia but distributed throughout the specialized wing cilia. We discuss this point in the manuscript (please see lines 235 – 239).

2. *RIC-8 and ODR-3 BIFC interaction: Authors use this clever strategy (with heat-shock RIC-8 to avoid developmental contributions) to show that RIC-8 and ODR-3 physically associate at specific regions of the AWC neuron (proximal region, ciliary base, NOT the entire wing). What is authors' interpretation? This would also be an appropriate discussion point.*

We observe the strongest BiFC signal at the cilia base and faint signal inside the AWC cilium (now outlined in Figure 4D). Since formation of the BiFC complex can be irreversible, we cannot distinguish the site of the initial interaction between RIC-8 and ODR-3 from the secondary location where the complex may have translocated after the initial binding event. Nonetheless, we did not consistently observe BiFC along the entire length of the AWC dendrite. This could be due to the fact that ODR-3 levels outside the cilia/cilia base are too low to be detected by BiFC, since ODR-3 is normally concentrated inside the cilia of AWC neurons (Roayaie et al., 1998 PMID: 9459442) (Figure 5A). Furthermore, the two proteins could be transported to cilia via distinct mechanisms/vesicles and therefore would not associate in the dendrite. We modified our conclusion for this experiment accordingly (please see lines 331-334).

3. *Authors show that RIC-8 but not AGS-3 regulates ODR-3 levels and propose that RIC-8 is a chaperone for ODR-3. The data supports this hypothesis/model. ODR-3 levels/localization are not changed in the ags-3 mutant, however this ODR-3 overexpression rescues ags-3 AWC morphogenesis defects. This result complicates authors' interpretation: "This result confirmed that cilia defects in ags-3 mutant are not due to reduced levels or mislocalization of ODR-3." Would authors please comment on this?*

Thank you, we clarified this conclusion (please see lines 412-413). We also expanded our interpretation of this result (please see lines 417-425).

4. *ODR-3 constitutively active overexpression phenotype (lines 361-363): “Interestingly, AWC distal dendrites…exhibited marked membranous extension at the ciliary base.” I agree but would be interested in hearing what authors think. It seems that this observation may relate to questions 1 and 2, which would be an appropriate discussion point*.

We think that the ectopic membrane projections in distal dendrites of animals over-expressing constitutively active ODR-3 are reminiscent of budding extracellular vesicles (EVs), which have been reported at the cilia base of sensory neurons (Wang et al., 2014 PMID: 24530063; Wang et al., 2021 PMID: 34270950; Razzauti and Laurent, 2021 PMID: 34533135). We now note this in the text of the manuscript (lines 453-455).

5. *If the wings are not required for AWC chemosensation, what is their purpose? I assumed, wrongly, that the wings were to increase surface area for odorants and other small molecules.*

Based on prior work in AWA neurons that also have morphologically complex cilia (please see lines 491-494), we suspect that AWC cilia morphology may be required for more nuanced sensory functions such as olfactory plasticity or habituation. We expanded our discussion on the future work that is needed to address the possible roles of the specialized AWC cilia in sensory biology (please see lines 497-500). Please also see response to Question 6 from Reviewer 3.

Reviewer #2
No concerns to address

Reviewer #3
1. *Line 112/113: Amphid neurons are not quite the majority of ciliated neurons in the hermaphrodite.*

Thank you, we corrected this statement (please see lines 113-114).

2. *Line 155: the extent of the rescue is only partial. Thus, can the authors truly discount a cell non-autonomous role for ric-8 in addition to the cell autonomous role that is outlined?*

Thank you for bringing this to our attention. Cilia phenotypes in *ric-8* hypomorphs are fully rescued when the full-length *ric-8* cDNA is expressed in all ciliated neurons under the *bbs-8* promoter (Fig. 3C), suggesting that RIC-8 functions in ciliated neurons. Lack of complete rescue with the AWC-specific *ceh-36Δ*p promoter could be due to sub-optimal levels or timing of the full-length RIC-8 expression under this promoter. Additionally, multiple RIC-8 isoforms are predicted to be expressed from the *ric-8* gene in *C. elegans* (WormBase); therefore, it is possible that other RIC-8 isoforms contribute to ciliogenesis in AWC. Overall, given that RIC-8 is expressed in AWC and that *ric-8*-dependent phenotypes in another ciliated neuron (AWA) are fully rescued by the pan-ciliary promoter collectively suggest that RIC-8 likely functions in AWC neurons to regulate ciliogenesis. However, it is true that we cannot completely rule out the possibility that RIC-8 may have a cell non-autonomous role in AWC ciliogenesis. We therefore toned down our conclusion for the rescue experiments accordingly (please see lines 154-159).

3. *Fig. 4 C-E: do the authors see any BiFC signal in the absence of a heat shock? I would have thought that is an important control to show?*

We would like to emphasize that our only goal for the BiFC experiment was to determine whether RIC-8 physically associates with ODR-3 *in vivo*. We did not include a no-heat-shock

condition as it would not have changed the interpretation of the experimental outcome. Specifically, if the heat-shock promoter were ‘leaky’ and drove RIC-8::VC expression at high enough levels to cause RIC-8-ODR-3 BiFC complex formation without heat shock, the conclusion of the experiment would have remained the same (i.e. RIC-8 and ODR-3 associate *in vivo*).

4. *Line 296: Although RIC-8::GFP is still localised in the AWC cilia of odr-3 mutants, the signal distribution, at least as presented in Fig 5C, looks different to the control (mutant seems to show accumulation of GFP at the cilia endings). This should be commented on.*

Thank you for bringing this to our attention. We replaced the image in panel 5C with a more representative image.

5. *The study looks at benzaldehyde chemoattraction in ags-3 and odr-3 mutants; why were ric-8 mutants not assessed (is this due to a locomotion defect in these worms ?).*

That is correct, *ric-8* mutants are paralyzed and therefore cannot be evaluated in behavioral assays (please see lines 504-507 and Miller et al., 2000 PMID: 10985349).

6. *Line 384-393: it is very interesting that significant cilium structure defects in worms do not necessarily have to mean cilium function defects. It would be useful to the reader to know if such instances have been observed in mammals, given that many mammalian studies measure things like cilium length, with the implication being that shorter or longer cilia likely have cilium function defects. Perhaps this is not necessarily always so as is being shown here in worms. Maybe there is a tolerance of cilium length change that does not affect at least some cilia functions. I realise this is a significant topic to address but perhaps a couple of extra lines of thought on this matter is warranted in the discussion.*

Thank you for this suggestion. We added an example from mammalian cone photoreceptors, which similarly to *C. elegans* wing neurons, possess morphologically complex cilia (please see lines 495-497).

7. *Figure 3A: indicate on the 1-566 schematic the positions of the GEF and the chaperone sequences.*

The exact boundaries of GEF/chaperone motifs in *C. elegans* RIC-8 have not been mapped.

8. *Figure 3C: why no FL rescue data for the md1909 mutant?*

We did not observe any phenotypic differences between *ric-8(md303)* and *ric-8(md1909)* alleles in our morphological analyses (please see lines 159-161); this is also consistent with other published studies that examined both alleles in other developmental contexts (e.g. Afshar et al., 2004 PMID: 15479639; Miller and Rand 2000 PMID: 11102364). Since AWA cilia defects in *ric-8(md303)* background are rescued upon re-expression of the full-length *ric-8* cDNA (Fig. 3B-C), we did not anticipate a different outcome in the *ric-8(md1909)* allele.

9. *Figure 4D: Interesting that the strongest BIFC signal is at the BB/PCMC region. Suggests that RIC-8 is preferentially binding ODR-3 outside the cilium, perhaps upstream of ODR-3s ciliary targeting? Can the authors comment on this?*

Please see our response to Question 2 from Reviewer 1.
